# Supplementary material for: Paenitracins, a novel family of bacitracin-type nonribosomal peptide antibiotics produced by plant-associated Paenibacillus species
Source: mSystems. 2026 Feb 17;11(3):e01496-25. doi: 10.1128/msystems.01496-25 (PMC13011432; doi:10.1128/msystems.01496-25)
Supplement: Supplemental Information — Supplemental figures and tables. [file msystems.01496-25-s0001.pdf]

Paenitracins: a novel family of bacitracin-type nonribosomal peptide antibiotics produced by plant-associated *Paenibacillus* species

Nataliia V. Machushynets[1,*], Somayah S. Elsayed[1], Chao Du[1], Vladyslav Lysenko[2], Mercedes de la Cruz[3], Pilar Sanchez[3], Olga Genilloud[3], Nathaniel I. Martin[2], Mark R. Liles[4], and Gilles P. van Wezel[1,5,*]

[1] Molecular Biotechnology Group, Leiden University, Institute of Biology, Leiden, The Netherlands

[2] Biological Chemistry Group, Leiden University, Institute of Biology, Leiden, The Netherlands

[3] Fundación MEDINA, Health Sciences Technology Park, Granada, Spain

[4] Department of Biological Sciences, Auburn University, Auburn, Alabama, USA

[5] Department of Microbial Ecology, Netherlands Institute of Ecology, Wageningen, The Netherlands

* Corresponding authors: Nataliia V. Machushynets, email: n.v.machushynets@biology.leidenuniv.nl; Gilles P. van Wezel, email: g.wezel@biology.leidenuniv.nl.

Table S1. Overview of 227 *Paenibacillus* spp. from the Auburn University Plant-Associated Microbial strain collection.

Isolate	Top hit	Pairwise similarity (%)	Crop	Rhizoplane/ Endophyte	Root #	City	State	Bioactivity against *E.coli* ATCC 25922	Bioactivity against *S. aureus* ATCC 29213
**JJ-6**	*P. xylanexedens*	99.39	Corn	Endophyte	1	Dunbar	Nebraska	–	–
**JJ-7**	*P. typhae*	98.70	Corn	Endophyte	1	Dunbar	Nebraska	–	–
**JJ-15**	*P. amylolyticus*	99.59	Corn	Endophyte	3	Dunbar	Nebraska	–	–
**JJ-16**	*P. peoriae*	99.59	Corn	Endophyte	3	Dunbar	Nebraska	+	–
**JJ-18***	*P. alginolyticus*	99.22	Corn	Endophyte	3	Dunbar	Nebraska	–	–
**JJ-21***	*P. peoriae*	99.66	Corn	Endophyte	3	Dunbar	Nebraska	+	–
**JJ-29**	*P. illinoisensis*	99.65	Corn	Endophyte	4	Dunbar	Nebraska	–	–
**JJ-33**	*P. lautus*	99.37	Corn	Endophyte	4	Dunbar	Nebraska	–	–
**JJ-39**	*P. terrigena*	99.25	Corn	Endophyte	6	Dunbar	Nebraska	–	–
**JJ-42**	*P. pectinilyticus*	98.78	Corn	Endophyte	6	Dunbar	Nebraska	–	–
**JJ-47**	*P. cineris*	100.00	Corn	Endophyte	7	Dunbar	Nebraska	–	–
**JJ-49**	*P. lautus*	99.16	Corn	Endophyte	7	Dunbar	Nebraska	–	–
**JJ-56**	*P. amylolyticus*	99.45	Corn	Endophyte	9	Dunbar	Nebraska	–	–
**JJ-59**	*P. aceris*	98.61	Corn	Endophyte	9	Dunbar	Nebraska	–	–
**JJ-60**	*P. etheri*	99.65	Corn	Endophyte	9	Dunbar	Nebraska	–	–
**JJ-72**	*P. amylolyticus*	99.80	Corn	Endophyte	12	Dunbar	Nebraska	–	–
**JJ-73**	*P. terrigena*	99.04	Corn	Endophyte	12	Dunbar	Nebraska	–	–
**JJ-77**	*P. aceris*	99.65	Corn	Endophyte	12	Dunbar	Nebraska	–	–
**JJ-90**	*P. typhae*	98.70	Corn	Endophyte	14	Dunbar	Nebraska	–	–
**JJ-92**	*P. lautus*	99.30	Corn	Endophyte	14	Dunbar	Nebraska	–	–
**JJ-93**	*P. aceris*	98.75	Corn	Endophyte	14	Dunbar	Nebraska	–	–
**JJ-95**	*P. aceris*	99.29	Corn	Endophyte	15	Dunbar	Nebraska	–	–
**JJ-99**	*P. aceris*	98.75	Corn	Endophyte	15	Dunbar	Nebraska	–	–
**JJ-102**	*P. xylanexedens*	99.39	Corn	Endophyte	16	Dunbar	Nebraska	–	–
**JJ-106**	*P. marinesediminis*	99.59	Soybean	Endophyte	1	Dunbar	Nebraska	–	–
**JJ-121**	*P. lautus*	99.23	Soybean	Endophyte	5	Dunbar	Nebraska	–	–
**JJ-148**	*P. aceris*	98.68	Soybean	Endophyte	11	Dunbar	Nebraska	–	–
**JJ-159**	*P. lautus*	99.23	Soybean	Endophyte	13	Dunbar	Nebraska	–	–
**JJ-174**	*P. amylolyticus*	99.25	Soybean	Endophyte	15	Dunbar	Nebraska	–	–
**JJ-180**	*P. aceris*	98.75	Soybean	Endophyte	16	Dunbar	Nebraska	–	–
**JJ-193**	*P. terrigena*	99.52	Corn	Endophyte	1	Dunbar	Nebraska	–	–
**JJ-194**	*P. amylolyticus*	99.32	Corn	Endophyte	3	Dunbar	Nebraska	–	–
**JJ-195**	*P. peoriae*	99.72	Corn	Endophyte	3	Dunbar	Nebraska	+	+
**JJ-216**	*P. pocheonensis*	99.04	Corn	Rhizoplane	1	Dunbar	Nebraska	–	–
**JJ-223**	*P. xylanilyticus*	98.37	Corn	Rhizoplane	1	Dunbar	Nebraska	–	–
**JJ-226**	*P. peoriae*	99.79	Corn	Rhizoplane	2	Dunbar	Nebraska	+	–
**JJ-227**	*P. peoriae*	99.79	Corn	Rhizoplane	2	Dunbar	Nebraska	+	+
**JJ-228**	*P. peoriae*	99.79	Corn	Rhizoplane	2	Dunbar	Nebraska	+	–
**JJ-231**	*P. illinoisensis*	99.86	Corn	Rhizoplane	3	Dunbar	Nebraska	–	–
**JJ-232**	*P. dongdonensis*	99.19	Corn	Rhizoplane	3	Dunbar	Nebraska	–	–

**JJ-235**	*P. amylolyticus*	99.66	Corn	Rhizoplane	3	Dunbar	Nebraska	–	–
**JJ-237**	*P. amylolyticus*	99.66	Corn	Rhizoplane	3	Dunbar	Nebraska	–	–
**JJ-246**	*P. oenotherae*	98.63	Corn	Rhizoplane	5	Dunbar	Nebraska	–	–
**JJ-253**	*P. pectinilyticus*	98.78	Corn	Rhizoplane	6	Dunbar	Nebraska	–	–
**JJ-268**	*P. tritici*	99.73	Corn	Rhizoplane	9	Dunbar	Nebraska	–	–
**JJ-270**	*P. tritici*	100.00	Corn	Rhizoplane	9	Dunbar	Nebraska	–	–
**JJ-272**	*P. tritici*	98.90	Corn	Rhizoplane	9	Dunbar	Nebraska	–	–
**JJ-287**	*P. agaridevorans*	97.41	Corn	Rhizoplane	13	Dunbar	Nebraska	–	–
**JJ-305**	*P. illinoisensis*	99.72	Corn	Rhizoplane	16	Dunbar	Nebraska	–	–
**JJ-310**	*P. endophyticus*	99.52	Soybean	Rhizoplane	1	Dunbar	Nebraska	–	–
**JJ-311**	*P. amylolyticus*	98.64	Soybean	Rhizoplane	1	Dunbar	Nebraska	–	–
**JJ-317**	*P. 'taohuashanense'*	99.86	Soybean	Rhizoplane	1	Dunbar	Nebraska	–	–
**JJ-324**	*P. lautus*	99.37	Soybean	Rhizoplane	2	Dunbar	Nebraska	–	–
**JJ-329**	*P. 'yonginensis'*	97.47	Soybean	Rhizoplane	3	Dunbar	Nebraska	–	–
**JJ-331**	*P. 'taohuashanense'*	98.66	Soybean	Rhizoplane	3	Dunbar	Nebraska	–	–
**JJ-332**	*P. catalpae*	99.59	Soybean	Rhizoplane	3	Dunbar	Nebraska	–	–
**JJ-333**	*P. jamilae*	99.86	Soybean	Rhizoplane	3	Dunbar	Nebraska	–	+
**JJ-337**	*P. illinoisensis*	99.79	Soybean	Rhizoplane	4	Dunbar	Nebraska	–	–
**JJ-338**	*P. lautus*	99.37	Soybean	Rhizoplane	4	Dunbar	Nebraska	–	–
**JJ-340**	*P. thiaminolyticus*	99.79	Soybean	Rhizoplane	4	Dunbar	Nebraska	–	–
**JJ-342**	*P. terrigena*	99.52	Soybean	Rhizoplane	4	Dunbar	Nebraska	–	–
**JJ-352**	*P. aceris*	98.61	Soybean	Rhizoplane	6	Dunbar	Nebraska	–	–
**JJ-355**	*P. illinoisensis*	99.72	Soybean	Rhizoplane	6	Dunbar	Nebraska	–	–
**JJ-364**	*P. urinalis*	99.35	Soybean	Rhizoplane	7	Dunbar	Nebraska	–	–
**JJ-365**	*P. odorifer*	99.51	Soybean	Rhizoplane	7	Dunbar	Nebraska	–	–
**JJ-372**	*P. aceris*	99.30	Soybean	Rhizoplane	8	Dunbar	Nebraska	–	–
**JJ-380**	*P. rigui*	98.18	Soybean	Rhizoplane	10	Dunbar	Nebraska	–	–
**JJ-385**	*P. silagei*	97.09	Soybean	Rhizoplane	11	Dunbar	Nebraska	–	–
**JJ-391**	*P. illinoisensis*	99.65	Soybean	Rhizoplane	12	Dunbar	Nebraska	–	–
**JJ-393**	*P. terrigena*	99.17	Soybean	Rhizoplane	13	Dunbar	Nebraska	–	–
**JJ-402**	*P. terrigena*	99.79	Soybean	Rhizoplane	14	Dunbar	Nebraska	–	–
**JJ-408**	*P. terrigena*	99.04	Soybean	Rhizoplane	15	Dunbar	Nebraska	–	–
**JJ-410**	*P. tritici*	99.93	Soybean	Rhizoplane	15	Dunbar	Nebraska	–	–
**JJ-411**	*P. aceris*	98.68	Soybean	Rhizoplane	15	Dunbar	Nebraska	–	–
**JJ-412**	*P. lautus*	99.23	Soybean	Rhizoplane	15	Dunbar	Nebraska	–	–
**JJ-413**	*P. 'taohuashanense'*	98.80	Soybean	Rhizoplane	16	Dunbar	Nebraska	–	–
**JJ-415**	*P. catalpae*	99.59	Soybean	Rhizoplane	16	Dunbar	Nebraska	–	–
**JJ-447**	*P. ehimensis*	96.56	Corn	Rhizoplane	12	Dunbar	Nebraska	–	–
**JJ-450**	*P. pectinilyticus*	99.20	Soybean	Rhizoplane	5	Dunbar	Nebraska	–	–
**JJ-460**	*P. pectinilyticus*	99.78	Corn	Endophyte	9	Dunbar	Nebraska	–	–
**JJ-467**	*P. pectinilyticus*	98.85	Soybean	Rhizoplane	5	Dunbar	Nebraska	–	–
**JJ-471**	*P. sabinae*	99.46	Soybean	Rhizoplane	15	Dunbar	Nebraska	–	–
**JJ-483**	*P. panacisoli*	99.65	Corn	Rhizoplane	2	Carrol	Iowa	–	–
**JJ-497**	*P. amylolyticus*	99.59	Corn	Rhizoplane	3	Carrol	Iowa	–	–
**JJ-514**	*P. dongdonensis*	99.12	Corn	Rhizoplane	4	Carrol	Iowa	–	–
**JJ-526**	*P. lautus*	99.21	Corn	Rhizoplane	6	Carrol	Iowa	–	–

**JJ-534**	*P. barcinonensis*	99.11	Corn	Rhizoplane	7	Carrol	Iowa	–	–
**JJ-539**	*P. cucumis*	100.00	Corn	Rhizoplane	8	Carrol	Iowa	–	–
**JJ-541**	*P. panacisoli*	99.72	Corn	Rhizoplane	8	Carrol	Iowa	–	–
**JJ-602**	*P. alvei*	99.19	Corn	Endophyte	5	Carrol	Iowa	–	–
**JJ-624**	*P. alkaliterrae*	99.33	Corn	Endophyte	7	Carrol	Iowa	–	–
**JJ-631**	*P. lautus*	99.09	Corn	Endophyte	8	Carrol	Iowa	–	–
**JJ-633**	*P. lautus*	99.09	Corn	Endophyte	8	Carrol	Iowa	–	–
**JJ-667**	*P. aceris*	98.68	Corn	Rhizoplane	3	Whitewater	Wisconsin	–	–
**JJ-676**	*P. 'taohuashanense'*	98.59	Corn	Rhizoplane	5	Whitewater	Wisconsin	–	–
**JJ-690**	*P. lautus*	99.58	Corn	Rhizoplane	7	Whitewater	Wisconsin	–	–
**JJ-702**	*P. tundrae*	99.46	Corn	Rhizoplane	8	Whitewater	Wisconsin	–	–
**JJ-718**	*P. 'taohuashanense'*	98.73	Corn	Rhizoplane	10	Whitewater	Wisconsin	–	–
**JJ-754**	*P. panacisoli*	99.59	Corn	Endophyte	5	Whitewater	Wisconsin	–	–
**JJ-764**	*P. castaneae*	97.70	Corn	Endophyte	6	Whitewater	Wisconsin	–	–
**JJ-773**	*P. amylolyticus*	99.59	Corn	Endophyte	7	Whitewater	Wisconsin	–	–
**JJ-778***	*P. amylolyticus*	99.80	Corn	Endophyte	8	Whitewater	Wisconsin	–	–
**JJ-779**	*P. lactis*	99.51	Corn	Endophyte	8	Whitewater	Wisconsin	–	–
**JJ-782**	*P. amylolyticus*	99.73	Corn	Endophyte	8	Whitewater	Wisconsin	–	–
**JJ-788**	*P. amylolyticus*	99.52	Corn	Endophyte	9	Whitewater	Wisconsin	–	–
**JJ-794**	*P. lautus*	99.44	Corn	Endophyte	10	Whitewater	Wisconsin	–	–
**JJ-798**	*P. 'taohuashanense'*	98.87	Corn	Endophyte	10	Whitewater	Wisconsin	–	–
**JJ-816**	*P. barcinonensis*	99.11	Corn	Rhizoplane	3	Carrol	Iowa	–	–
**JJ-820**	*P. tundrae*	99.59	Corn	Rhizoplane	6	Carrol	Iowa	–	–
**JJ-824**	*P. illinoisensis*	99.86	Corn	Rhizoplane	9	Carrol	Iowa	–	–
**JJ-845**	*P. jamilae*	99.52	Corn	Endophyte	10	Whitewater	Wisconsin	+	–
**JJ-870**	*P. castaneae*	99.32	Soybean	Rhizoplane	4	Whitewater	Wisconsin	–	–
**JJ-890**	*P. contaminans*	99.93	Soybean	Rhizoplane	7	Whitewater	Wisconsin	–	–
**JJ-955**	*P. silagei*	98.84	Soybean	Endophyte	7	Whitewater	Wisconsin	–	–
**JJ-960**	*P. aestuarii*	95.04	Soybean	Endophyte	8	Whitewater	Wisconsin	–	–
**JJ-961**	*P. marinisediminis*	99.52	Soybean	Endophyte	8	Whitewater	Wisconsin	–	–
**JJ-963**	*P. tritici*	99.57	Soybean	Endophyte	8	Whitewater	Wisconsin	–	–
**JJ-964**	*P. lautus*	99.44	Soybean	Endophyte	8	Whitewater	Wisconsin	–	–
**JJ-985**	*P. castaneae*	99.39	Soybean	Rhizoplane	2	Whitewater	Wisconsin	–	–
**JJ-1000**	*P. xylanexedens*	99.86	Corn	Rhizoplane	1	Sparta	Illinois	–	–
**JJ-1004**	*P. barcinonensis*	98.69	Corn	Rhizoplane	1	Sparta	Illinois	–	–
**JJ-1057**	*P. amylolyticus*	99.80	Corn	Rhizoplane	8	Sparta	Illinois	–	–
**JJ-1059**	*P. lautus*	99.23	Corn	Rhizoplane	8	Sparta	Illinois	–	–
**JJ-1069**	*P. barcinonensis*	98.69	Corn	Endophyte	1	Sparta	Illinois	–	–
**JJ-1074**	*P. amylolyticus*	99.86	Corn	Endophyte	1	Sparta	Illinois	–	–
**JJ-1099**	*P. odorifer*	98.58	Corn	Endophyte	6	Sparta	Illinois	–	–
**JJ-1103**	*P. xylanilyticus*	100.00	Corn	Endophyte	6	Sparta	Illinois	–	–
**JJ-1115**	*P. amylolyticus*	99.59	Corn	Endophyte	9	Sparta	Illinois	–	–
**JJ-1181**	*P. terrigena*	99.17	Soybean	Rhizoplane	8	Sparta	Illinois	–	–
**JJ-1187**	*P. illinoisensis*	99.79	Soybean	Rhizoplane	8	Sparta	Illinois	–	–
**JJ-1277**	*P. dongdonensis*	99.12	Corn	Rhizoplane	5	Sparta	Illinois	–	–
**JJ-1283**	*P. pocheonensis*	98.97	Corn	Endophyte	9	Sparta	Illinois	–	–

**JJ-1311**	*P. amylolyticus*	99.58	Soybean	Endophyte	6	Sparta	Illinois	–	–
**JJ-1319**	*P. barcinonensis*	99.17	Soybean	Rhizoplane	2	Carrol	Iowa	–	–
**JJ-1343**	*P. barcinonensis*	99.21	Soybean	Rhizoplane	5	Carrol	Iowa	–	–
**JJ-1371**	*P. catalpae*	99.66	Soybean	Rhizoplane	9	Carrol	Iowa	–	–
**JJ-1402**	*P. susongensis*	99.17	Soybean	Endophyte	2	Carrol	Iowa	–	–
**JJ-1405**	*P. susongensis*	99.17	Soybean	Endophyte	2	Carrol	Iowa	–	–
**JJ-1410**	*P. odorifer*	98.78	Soybean	Endophyte	3	Carrol	Iowa	–	–
**JJ-1415**	*P. selenitireducens*	99.23	Soybean	Endophyte	4	Carrol	Iowa	–	–
**JJ-1425**	*P. granivorans*	96.71	Soybean	Endophyte	5	Carrol	Iowa	–	–
**JJ-1461**	*P. barcinonensis*	99.24	Corn	Rhizoplane	1	Troy	Ohio	–	–
**JJ-1465**	*P. cucumis*	99.93	Corn	Rhizoplane	1	Troy	Ohio	–	–
**JJ-1470**	*P. amylolyticus*	99.45	Corn	Rhizoplane	2	Troy	Ohio	–	–
**JJ-1476**	*P. massiliensis*	99.86	Corn	Rhizoplane	2	Troy	Ohio	–	–
**JJ-1487**	*P. pocheonensis*	98.97	Corn	Rhizoplane	3	Troy	Ohio	–	–
**JJ-1501**	*P. lautus*	99.37	Corn	Rhizoplane	6	Troy	Ohio	–	–
**JJ-1516**	*P. susongensis*	99.24	Corn	Rhizoplane	8	Troy	Ohio	–	–
**JJ-1522**	*P. barcinonensis*	99.31	Corn	Endophyte	2	Troy	Ohio	–	–
**JJ-1524**	*P. tritici*	99.71	Corn	Endophyte	2	Troy	Ohio	–	–
**JJ-1525**	*P. odorifer*	99.93	Corn	Endophyte	2	Troy	Ohio	–	–
**JJ-1531**	*P. rhizoplanae*	98.21	Corn	Endophyte	3	Troy	Ohio	–	–
**JJ-1544**	*P. barcinonensis*	99.17	Corn	Endophyte	5	Troy	Ohio	–	–
**JJ-1549**	*P. massiliensis*	99.73	Corn	Endophyte	5	Troy	Ohio	–	–
**JJ-1553**	*P. cucumis*	99.79	Corn	Endophyte	6	Troy	Ohio	–	–
**JJ-1555**	*P. barcinonensis*	99.31	Corn	Endophyte	6	Troy	Ohio	–	–
**JJ-1564**	*P. cucumis*	99.59	Corn	Endophyte	7	Troy	Ohio	–	–
**JJ-1570**	*P. susongensis*	99.17	Corn	Endophyte	8	Troy	Ohio	–	–
**JJ-1580**	*P. peoriae*	99.72	Soybean	Rhizoplane	1	Troy	Ohio	+	–
**JJ-1582**	*P. peoriae*	99.65	Soybean	Rhizoplane	1	Troy	Ohio	–	–
**JJ-1586**	*P. cucumis*	100.00	Soybean	Rhizoplane	2	Troy	Ohio	–	–
**JJ-1587**	*P. illinoisensis*	99.72	Soybean	Rhizoplane	2	Troy	Ohio	–	–
**JJ-1588**	*P. amylolyticus*	99.52	Soybean	Rhizoplane	2	Troy	Ohio	–	–
**JJ-1591**	*P. taichungensis*	99.73	Soybean	Rhizoplane	2	Troy	Ohio	–	–
**JJ-1595**	*P. odorifer*	98.48	Soybean	Rhizoplane	3	Troy	Ohio	–	–
**JJ-1596**	*P. barcinonensis*	99.17	Soybean	Rhizoplane	3	Troy	Ohio	–	–
**JJ-1598**	*P. odorifer*	99.80	Soybean	Rhizoplane	3	Troy	Ohio	–	–
**JJ-1601**	*P. illinoisensis*	99.79	Soybean	Rhizoplane	3	Troy	Ohio	–	–
**JJ-1602**	*P. typhae*	98.18	Soybean	Rhizoplane	3	Troy	Ohio	–	–
**JJ-1603**	*P. peoriae*	99.86	Soybean	Rhizoplane	3	Troy	Ohio	+	–
**JJ-1604**	*P. polymyxa*	100.00	Soybean	Rhizoplane	3	Troy	Ohio	+	–
**JJ-1611**	*P. typhae*	97.08	Soybean	Rhizoplane	4	Troy	Ohio	–	–
**JJ-1612**	*P. barcinonensis*	99.24	Soybean	Rhizoplane	4	Troy	Ohio	–	–
**JJ-1614**	*P. peoriae*	99.65	Soybean	Rhizoplane	4	Troy	Ohio	+	–
**JJ-1615**	*P. typhae*	99.45	Soybean	Rhizoplane	4	Troy	Ohio	–	–
**JJ-1620**	*P. massiliensis*	99.86	Soybean	Rhizoplane	5	Troy	Ohio	–	–
**JJ-1623**	*P. dongdonensis*	99.12	Soybean	Rhizoplane	5	Troy	Ohio	–	–
**JJ-1631**	*P. dongdonensis*	99.27	Soybean	Rhizoplane	6	Troy	Ohio	–	–
**JJ-1638**	*P. peoriae*	99.72	Soybean	Rhizoplane	7	Troy	Ohio	+	+

Classified as | Public - Publiek (Openbaar)

**JJ-1639**	*P. cucumis*	100.00	Soybean	Rhizoplane	7	Troy	Ohio	–	–
**JJ-1640**	*P. peoriae*	99.72	Soybean	Rhizoplane	7	Troy	Ohio	+	–
**JJ-1648**	*P. turicensis*	97.36	Soybean	Rhizoplane	8	Troy	Ohio	–	–
**JJ-1650**	*P. jamilae*	99.88	Soybean	Rhizoplane	8	Troy	Ohio	+	–
**JJ-1652**	*P. peoriae*	99.72	Soybean	Rhizoplane	9	Troy	Ohio	+	–
**JJ-1653**	*P. cucumis*	100.00	Soybean	Rhizoplane	9	Troy	Ohio	–	–
**JJ-1667**	*P. odorifer*	99.86	Soybean	Endophyte	1	Troy	Ohio	–	–
**JJ-1669**	*P. odorifer*	98.58	Soybean	Endophyte	1	Troy	Ohio	–	–
**JJ-1678**	*P. cucumis*	100.00	Soybean	Endophyte	2	Troy	Ohio	–	–
**JJ-1679**	*P. cucumis*	100.00	Soybean	Endophyte	2	Troy	Ohio	–	–
**JJ-1680**	*P. massiliensis*	99.86	Soybean	Endophyte	2	Troy	Ohio	–	–
**JJ-1683***	*P. jamilae*	99.73	Soybean	Endophyte	3	Troy	Ohio	+	–
**JJ-1684**	*P. cucumis*	99.31	Soybean	Endophyte	3	Troy	Ohio	–	–
**JJ-1688**	*P. massiliensis*	99.86	Soybean	Endophyte	3	Troy	Ohio	–	–
**JJ-1692**	*P. typhae*	99.59	Soybean	Endophyte	4	Troy	Ohio	–	–
**JJ-1693**	*P. amylolyticus*	99.52	Soybean	Endophyte	4	Troy	Ohio	–	–
**JJ-1701**	*P. typhae*	99.66	Soybean	Endophyte	5	Troy	Ohio	–	–
**JJ-1703**	*P. typhae*	99.59	Soybean	Endophyte	5	Troy	Ohio	–	–
**JJ-1709**	*P. massiliensis*	99.93	Soybean	Endophyte	5	Troy	Ohio	–	–
**JJ-1715**	*P. peoriae*	99.72	Soybean	Endophyte	6	Troy	Ohio	+	+
**JJ-1720**	*P. peoriae*	99.72	Soybean	Endophyte	6	Troy	Ohio	+	+
**JJ-1722***	*P. peoriae*	99.72	Soybean	Endophyte	7	Troy	Ohio	+	+
**JJ-1723**	*P. dongdonensis*	99.27	Soybean	Endophyte	7	Troy	Ohio	–	–
**JJ-1724**	*P. peoriae*	99.72	Soybean	Endophyte	7	Troy	Ohio	+	+
**JJ-1725**	*P. amylolyticus*	99.66	Soybean	Endophyte	7	Troy	Ohio	–	–
**JJ-1729**	*P. polymyxa*	100.00	Soybean	Endophyte	8	Troy	Ohio	–	–
**JJ-1736**	*P. typhae*	99.59	Soybean	Endophyte	8	Troy	Ohio	–	–
**JJ-1742**	*P. cucumis*	100.00	Soybean	Endophyte	9	Troy	Ohio	–	–
**JJ-1743**	*P. polymyxa*	99.93	Soybean	Endophyte	9	Troy	Ohio	+	–
**JJ-1744**	*P. typhae*	99.65	Soybean	Endophyte	9	Troy	Ohio	–	–
**JJ-1747**	*P. polymyxa*	99.65	Soybean	Endophyte	10	Troy	Ohio	+	+
**JJ-1755**	*P. graminis*	100.00	Soybean	Rhizoplane	5	Carrol	Iowa	–	–
**JJ-1759**	*P. catalpae*	99.66	Soybean	Rhizoplane	8	Carrol	Iowa	–	–
**JJ-1762**	*P. alginolyticus*	99.29	Soybean	Endophyte	4	Carrol	Iowa	–	–
**JJ-1774**	*P. susongensis*	99.21	Corn	Rhizoplane	2	Troy	Ohio	–	–
**JJ-1781**	*P. barcinonensis*	99.03	Corn	Rhizoplane	5	Troy	Ohio	–	–
**JJ-1783**	*P. sonchi*	98.64	Corn	Rhizoplane	6	Troy	Ohio	–	–
**JJ-1789**	*P. dongdonensis*	99.27	Soybean	Rhizoplane	2	Troy	Ohio	–	–
**JJ-1817**	*P. rhizoplanae*	98.35	Corn	Endophyte	6	Troy	Ohio	–	–
**JJ-1819**	*P. contaminans*	99.93	Soybean	Endophyte	1	Troy	Ohio	–	–
**JJ-1831**	*P. typhae*	97.33	Soybean	Endophyte	9	Troy	Ohio	–	–
**JM-929**	*P. odorifer*	99.51	Cotton	Endophyte	n.a	Tallassee	Alabama	–	–
**JM-1337**	*P. cucumis*	99.86	n.a	Endophyte	n.a	Tallassee	Alabama	–	–
**JM-1355**	*P. barcinonensis*	99.16	n.a	Endophyte	n.a	Tallassee	Alabama	–	–
**JM-1368**	*P. barcinonensis*	99.07	n.a	Endophyte	n.a	Tallassee	Alabama	–	–
**JM-1419**	*P. cucumis*	99.86	n.a	Endophyte	n.a	Tallassee	Alabama	–	–
**JM-1420**	*P. cucumis*	99.86	n.a	Endophyte	n.a	Tallassee	Alabama	–	–

**AP-66**	*P. silvae*	99.50	n.a	n.a	n.a	n.a	n.a	–	–

* Whole genome sequence is available

Table S2. List of *Paenibacillus* type strains used for the phylogenetic tree construction.

Strain	GenBank accession number
P. massiliensis subsp. panacisoli DSM 21345	AB245384.1
P. ehimensis DSM 11029	AY116665.1
P. illinoisensis DSM 11733	NR_115624.1
P. selenitireducens KCTC 33157	NR_133807.1
P. cucumis DSM 101601	NR_149778.1
P. thiaminolyticus DSM 7262	AB073197.1
P. terrigena DSM 21567	AB248087.1
P. granivorans	AF237682.1
P. turicensis DSM 14349	AF378694.1
P. agaridevorans DSM 1355	AJ345023.1
P. cineris DSM 16945	AJ575658.1
P. barcinonensis DSM 15478	AJ716019.1
P. lactis DSM 15596	AY257868.1
P. xylanilyticus DSM 17255	AY427832.1
P. alkaliterrae DSM 17040	AY960748.1
P. urinalis DSM 22281	EF212892.1
P. contaminans BCRC 17728	EF626690.1
P. taichungensis DSM 19942	EU179327.1
P. peoriae DSM 8320	EU391157.1
P. xylanexedens DSM 21292	EU558281.1
P. tundrae DSM 21291	EU558284.1
P. typhae DSM 25190	NR_109462.1
P. pocheonensis DSM 23906	NR_112565.1
P. alginolyticus DSM 5050	NR_115595.1
P. sonchi CCBAU 83901	NR_115751.1
P. aestuarii DSM 23861	NR_116365.1
P. catalpae DSM 24714	NR_118012.1
P. taohuashanense DSM 25809	NR_118393.1
P. sabinae T27	NR_121732.2
P. dongdonensis DSM 27607	NR_134112.1
P. susongensis JCM 19951	NR_134118.1
P. endophyticus LMG 27297	NR_135705.1
P. oenotherae JCM 19573	NR_136822.1
P. etheri DSM 29760	NR_148622.1
P. aceris DSM 24950	NR_156841.1
P. rhizoplanae DSM 103963	NR_156842.1
P. tritici CECT 9125	NR_157638.1
P. solisilvae JCM 32513	NR_175458.1
P. lautus DSM 3035	NZ_BIMF01000051.1
P. amylolyticus NBRC 15957	NZ_BIMJ01000009.1
P. rigui WPCB173	NZ_NMQW01000006.1

Table S3. Annotation of the known specialized metabolites detected in the extracts of 227 plant-associated *Paenibacillus* isolates. For the metabolites highlighted in bold, annotation was performed on the MS/MS level.

Parent ion *m/z*	Adduct ion	Compound	Family	Bioactivity
442.2839	$[M+2H]^{2+}$	Fusaricidin A	Fusaricidins	Gram +, Fungi (Kajimura & Kaneda, 1996)
**449.2909**	$[M+2H]^{2+}$	Fusaricidin B/Antibiotic LI-F05A/LI-F06A	Fusaricidins	Gram +, Fungi (Ryu *et al.*, 2017)
449.2921	$[M+2H]^{2+}$	Fusaricidin B/Antibiotic LI-F05A/LI-F06A	Fusaricidins	Gram +, Fungi (Ryu *et al.*, 2017)
456.300	$[M+2H]^{2+}$	Antibiotic LI-F05B/LI-F06B/LI-F08A	Fusaricidins	Gram +, Fungi (Ryu *et al.*, 2017)
463.3076	$[M+2H]^{2+}$	Antibiotic LI-F08B	Fusaricidins	Gram +, Fungi (Ryu *et al.*, 2017)
463.3077	$[M+2H]^{2+}$	Antibiotic LI-F08B	Fusaricidins	Gram +, Fungi (Ryu *et al.*, 2017)
466.2835	$[M+2H]^{2+}$	Antibiotic LI-F07A	Fusaricidins	Gram +, Fungi (Kajimura & Kaneda, 1996)
466.2848	$[M+2H]^{2+}$	Antibiotic LI-F07A	Fusaricidins	Gram +, Fungi (Kajimura & Kaneda, 1996)
473.2915	$[M+2H]^{2+}$	Antibiotic LI-F07B	Fusaricidins	Gram +, Fungi (Ryu *et al.*, 2017)
481.2895	$[M+2H]^{2+}$	Fusaricidine D	Fusaricidins	Gram +, Fungi (Ryu *et al.*, 2017)
883.5606	$[M+H]^{+}$	Fusaricidin A	Fusaricidins	Gram +, Fungi (Kajimura & Kaneda, 1996)
911.5916	$[M+H]^{+}$	Antibiotic LI-F08A	Fusaricidins	Gram +, Fungi (Kajimura & Kaneda, 1996)
911.5921	$[M+H]^{+}$	Antibiotic LI-F08A	Fusaricidins	Gram +, Fungi (Kajimura & Kaneda, 1996)
925.6076	$[M+H]^{+}$	Antibiotic LI-F08B	Fusaricidins	Gram +, Fungi (Ryu *et al.*, 2017)
945.5767	$[M+H]^{+}$	Antibiotic LI-F07B	Fusaricidins	Gram +, Fungi (Ryu *et al.*, 2017)
947.5557	$[M+H]^{+}$	Fusaricidin C	Fusaricidins	Gram +, Fungi

				(Kajimura & Kaneda, 1996)
961.5712	$[M+H]^{+}$	Fusaricidine D	Fusaricidins	Gram +, Fungi (Ryu *et al.*, 2017)
961.5718	$[M+H]^{+}$	Fusaricidine D	Fusaricidins	Gram +, Fungi (Ryu *et al.*, 2017)
381.9119	$[M+3H]^{3+}$	Polymyxin M2/A1	Polymyxins	Gram – (Martin *et al.*, 2003)
385.9241	$[M+3H]^{3+}$	Polymyxin E	Polymyxins	Gram – (Ikai *et al.*, 1998)
386.5838	$[M+3H]^{3+}$	Polymyxin M1/A2	Polymyxins	Gram – (Martin *et al.*, 2003)
386.5839	$[M+3H]^{3+}$	Polymyxin M1/A2	Polymyxins	Gram – (Martin *et al.*, 2003)
390.5958	$[M+3H]^{3+}$	Polymyxin E	Polymyxins	Gram – (Ikai *et al.*, 1998)
**390.596**	$[M+3H]^{3+}$	Polymyxin E	Polymyxins	Gram – (Ikai *et al.*, 1998)
397.9119	$[M+3H]^{3+}$	Polymyxin P1	Polymyxins	Gram – (Niu *et al.*, 2013)
401.9238	$[M+3H]^{3+}$	Polymyxin B	Polymyxins	Gram – (Pittenauer *et al.*, 2006)
579.371	$[M+2H]^{2+}$	Polymyxin M1	Polymyxins	Gram – (Martin *et al.*, 2003)
579.3713	$[M+2H]^{2+}$	Polymyxin M1	Polymyxins	Gram – (Martin *et al.*, 2003)
585.3898	$[M+2H]^{2+}$	Polymyxin E7	Polymyxins	Gram – (Ikai *et al.*, 1998)
596.3656	$[M+2H]^{2+}$	Polymyxin P1	Polymyxins	Gram – (Niu *et al.*, 2013)
602.382	$[M+2H]^{2+}$	Polymyxin B5	Polymyxins	Gram – (Pittenauer *et al.*, 2006)
610.3812	$[M+2H]^{2+}$	Polymyxin B6	Polymyxins	Gram – (Pittenauer *et al.*, 2006)
486.9491	$[M+3H]^{3+}$	Tridecaptin B_1	Tridecaptins	Gram – (Cochrane *et al.*, 2015)
**496.9524**	$[M+3H]^{3+}$	Tridecaptin M	Tridecaptins	Gram –

				(Jangra *et al.*, 2019b)
744.9239	$[M+2H]^{2+}$	Tridecaptin M	Tridecaptins	Gram –<br>(Jangra *et al.*, 2019b)
789.9477	$[M+2H]^{2+}$	Tridecaptin A_3	Tridecaptins	Gram –<br>(Lohans *et al.*, 2012)
539.9715	$[M+3H]^{3+}$	Tridecaptin A_5	Tridecaptins	Gram -, Gram +

Table S4. NMR data of paenitracin A (1), measured in DMSO-d_6 at 298 K*.

	Residue	NH	$H_\alpha(C_\alpha$, type)	$H_\beta(C_\beta$, type)	Other
**1**	**1-Ile**	ND	3.55,d (57.0, CH)	1.66 (39.5, CH)	1 C: ND<br>4 CH_2: 1.41, 1.15 (25.5)<br>5 CH_3: 0.84 (11.3)<br>6 CH_3: 0.81 (13.3)
**2**	**2-Cys**		5.13 (78.5, CH)	3.41 (32.7, CH_2)	
**3**	**3-Leu**	7.75	4.35 (51.9, CH)	1.52, 1.46 (41.0, CH_2)	4 CH: 1.54 (24.0)<br>5 CH_3: 0.85 (21.2)<br>6 CH_3: 0.89 (22.8)
**4**	**4-Glu**	8.18	4.06 (53.6, CH)	1.81, 1.75 (27.7, CH_2)	4 CH_2: 2.19 (32.8, CH_2)
**5**	**5-Ile**	7.22	3.95 (57.6, CH)	1.75 (35.4, CH)	4 CH_2: 1.46, 1.17 (24.3)<br>5 CH_3: 0.78 (10.8)<br>6 CH_3: 0.94 (15.0)
**6**	**6-Lys**	8.46	4.49 (52.9, CH)	1.42, 1.15 (25.5, CH_2)	4 CH_2: 1.54 (24.0)<br>5 CH_2: 1.47 (24.3)<br>6 CH_2: 2.95, 2.82 (36.8)<br>NH: 7.52
**7**	**7-Leu**	10.37	4.22 (49.8, CH)	1.61, 1.36 (38.7, CH_2)	4 CH: 1.51 (24.1)<br>5 CH_3: 0.77 (20.4)<br>6 CH_3: 0.85 (22.9)
**8**	**8-Ile**	7.09	4.26 (55.6, CH)	1.68 (37.5, CH)	4 CH_2: 1.28, 0.70 (23.9)<br>5 CH_3: 0.70 (11.5)<br>6 CH_3: 0.66 (14.1)
**9**	**9-Trp**	8.98	5.18 (52.2, CH)	3.11, 2.83 (28.5, CH_2)	4 C: ND<br>5 CH: 7.18 (123.2)<br>6 NH: 10.9<br>7 C: 135.5<br>8 CH: 7.26 (110.7)<br>9 CH: 7.01 (120.3)<br>10 CH: 6.95 (117.7)<br>11 CH: 7.48 (117.8)<br>12 C: 126.7
**10**	**10-Thr**	10.78	3.66 (60.4, CH)	4.02 (66.8, CH)	4 CH_3: 0.67 (18.5)
**11**	**11-Asp**	8.84	4.09 (50.2, CH)	2.43, 2.01 (38.2, CH_2)	
**12**	**12-Asn**	7.82	3.94 (51.8, CH)	2.38, 2.18 (35.2, CH_2)	NH_2: 7.58, 6.74

* ^{1}H 850 MHz and ^{13}C chemical shifts inferred from HSQC and HMBC spectra

ND: not determined under these experimental conditions (and also all carbonyl carbons).

Table S5. Retention times (t_R, min) of the L-FDAA derivatives for natural paenitracin A (**1**) and standard amino acids.

	**[M+H]⁺**	**t_R, min**			
		L-AA (standard)	D-AA (standard)	Paen A	Stereochemical assignment
**Asp**	386.0943	4.75	4.86	4.75/4.86	**L + D**
**Cys***	626.126	6.37	6.62	-	**NA**
**Glu**	400.1099	4.92	5.07	5.07	**D**
**Ile**	384.1514	6.40	6.93	6.40	**L**
**Leu**	384.1514	6.50	7.01	6.50/7.01	**L + D**
**Lys***	651.2118	6.45	6.63	6.45	**L**
**Thr**	372.115	4.75	5.12	4.75	**L**
**Trp**	457.1466	6.31	6.58	-	**NA**

* Product of double-addition of Marfey`s reagent

NA: not available

Table S6. Retention times (t_R, min) of the L-FDAA derivatives for natural paenitracin B (**2**) and standard amino acids.

	**[M+H]⁺**	**t_R, min**			
		L-AA (standard)	D-AA (standard)	Paen B	Stereochemical assignment
**Asp**	386.0943	4.75	4.86	4.75/4.86	**L + D**
**Cys***	626.126	6.37	6.62	-	**NA**
**Glu**	400.1099	4.92	5.07	5.07	**D**
**Ile**	384.1514	6.40	6.93	6.40	**L**
**Leu**	384.1514	6.50	7.01	6.50/7.01	**L + D**
**Lys***	651.2118	6.45	6.63	6.45	**L**
**Thr**	372.115	4.75	5.12	4.75	**L**
**Trp**	457.1466	6.31	6.58	-	**NA**

* Product of double-addition of Marfey`s reagent

NA: not available

Table S7. Antagonization of in vitro antibacterial activity of paenitracin A against *S. aureus* ATCC 29213 by addition of C_{10}PP.

Antagonist	$ZnSO_4$	*S. aureus* ATCC 29213
**C_{10}PP**	0.3 mM	G
**-**	0.3 mM	NG
**C_{10}PP**	-	G
**-**	-	G

Experiments were performed in triplicates. NG = no visible bacterial growth, corresponding to unaffected antibiotic activity. G = visible bacterial growth, corresponding to antagonization of antibiotic activity.

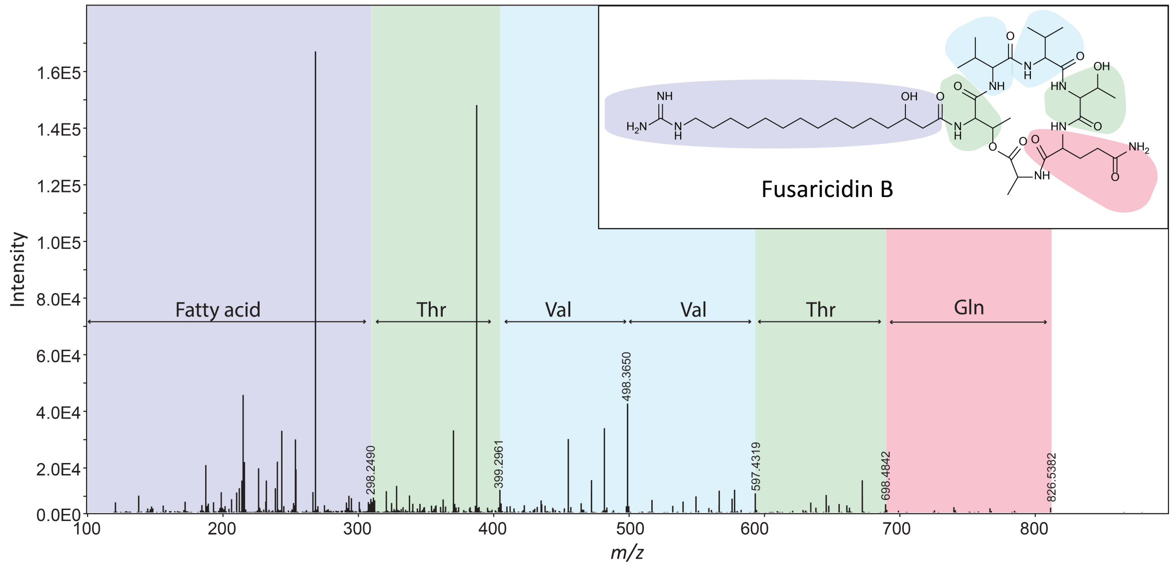

Figure S1. MS/MS spectrum of fusaricidin B (precursor ion $[M + 2H]^{2+}$ *m/z* 449.2909). The assignment of the sequence of amino acid residues is based on the mass differences between the consecutive *b* ions.

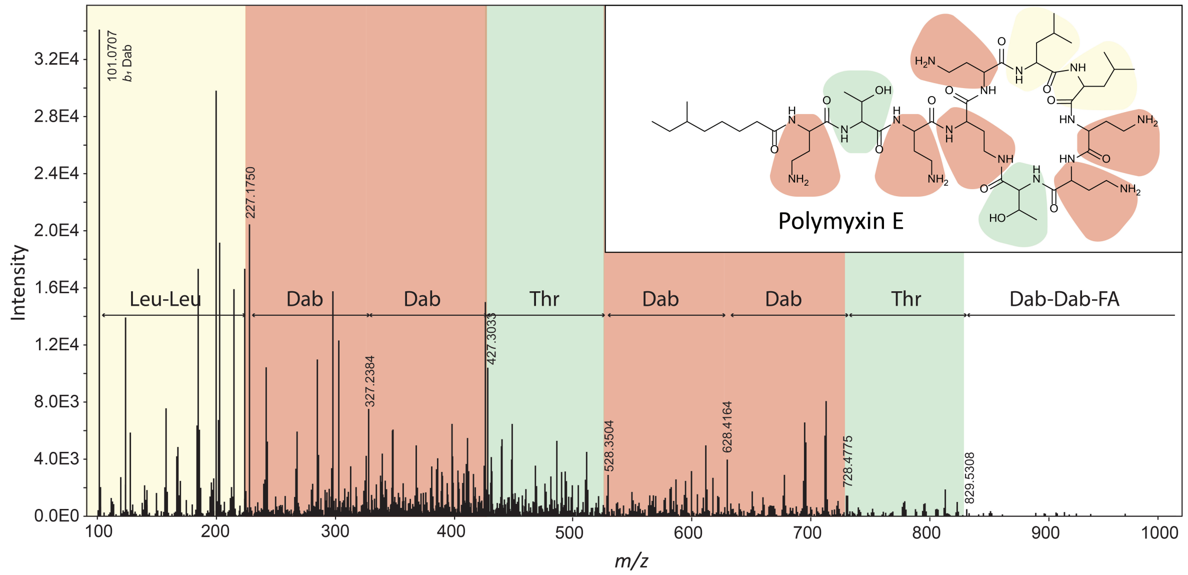

Figure S2. MS/MS spectrum of polymyxin E (precursor ion $[M + 3H]^{3+}$ *m/z* 390.596). The assignment of the sequence of amino acid residues is based on the mass differences between the consecutive *b* ions.

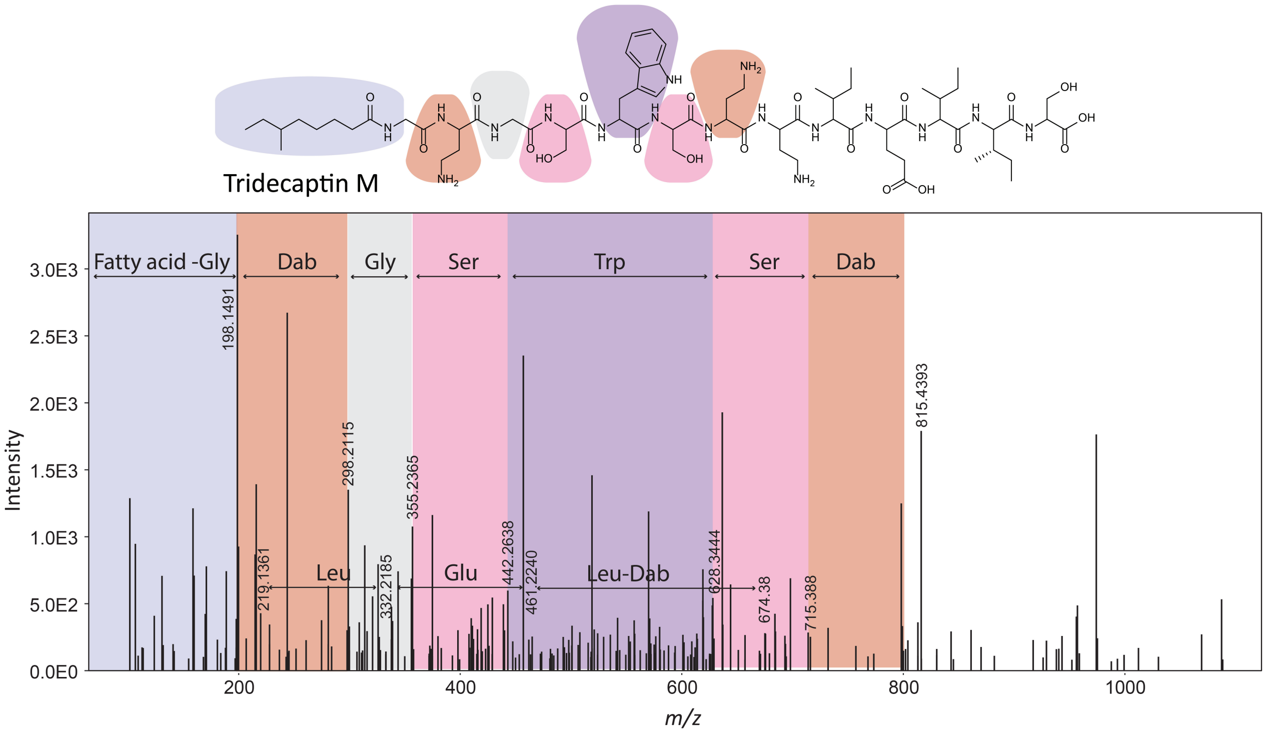

Figure S3. MS/MS spectrum of tridecaptin M (precursor ion $[M + 3H]^{3+}$ *m/z* 496.9524). The assignment of the sequence of amino acid residues is based on the mass differences between the consecutive *b* ions.

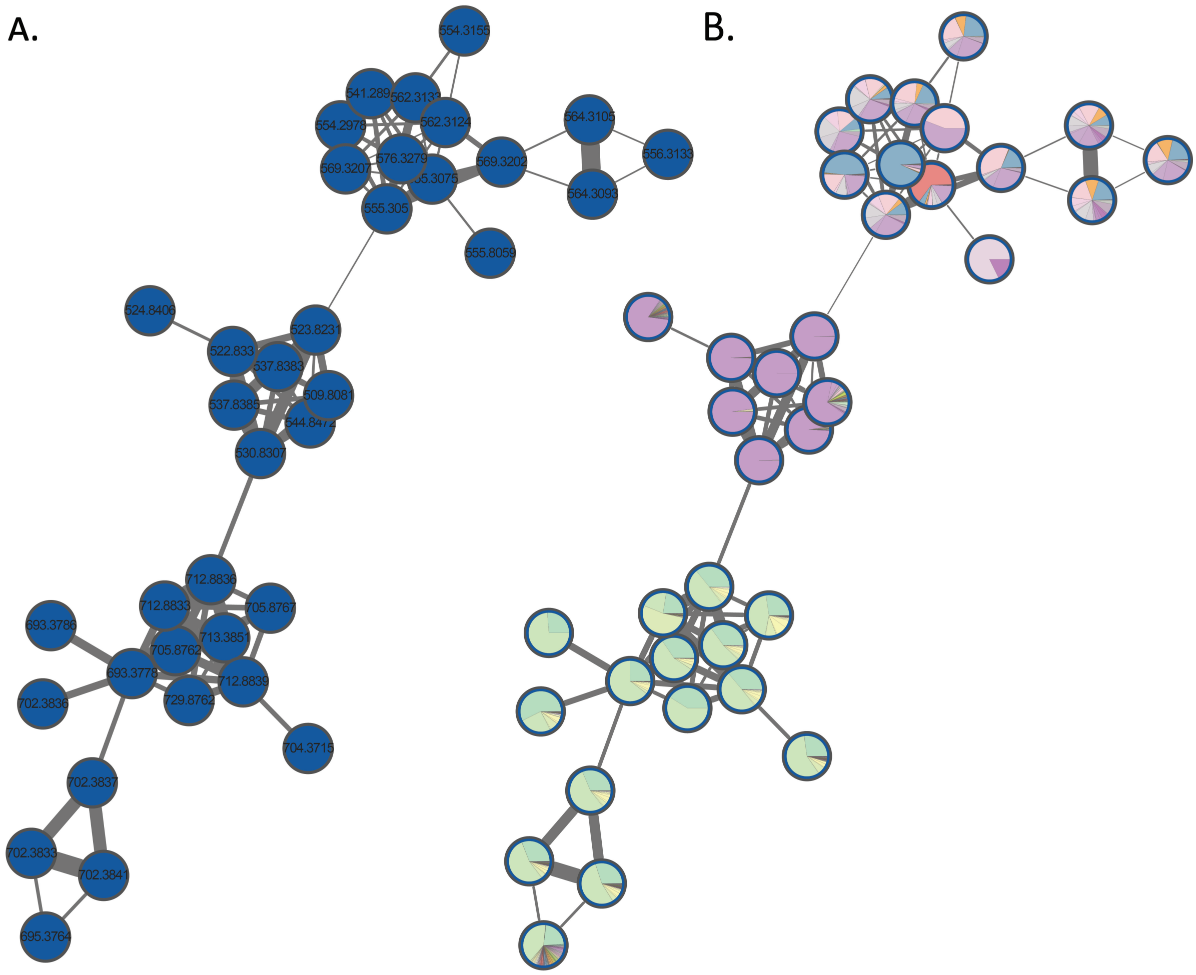

Figure S4. A. The MassQL-annotated molecular family of lysine-containing natural products. **B**. On the same molecular family, pie charts were mapped to the nodes to represent the relative precursor ion intensities within each extract.

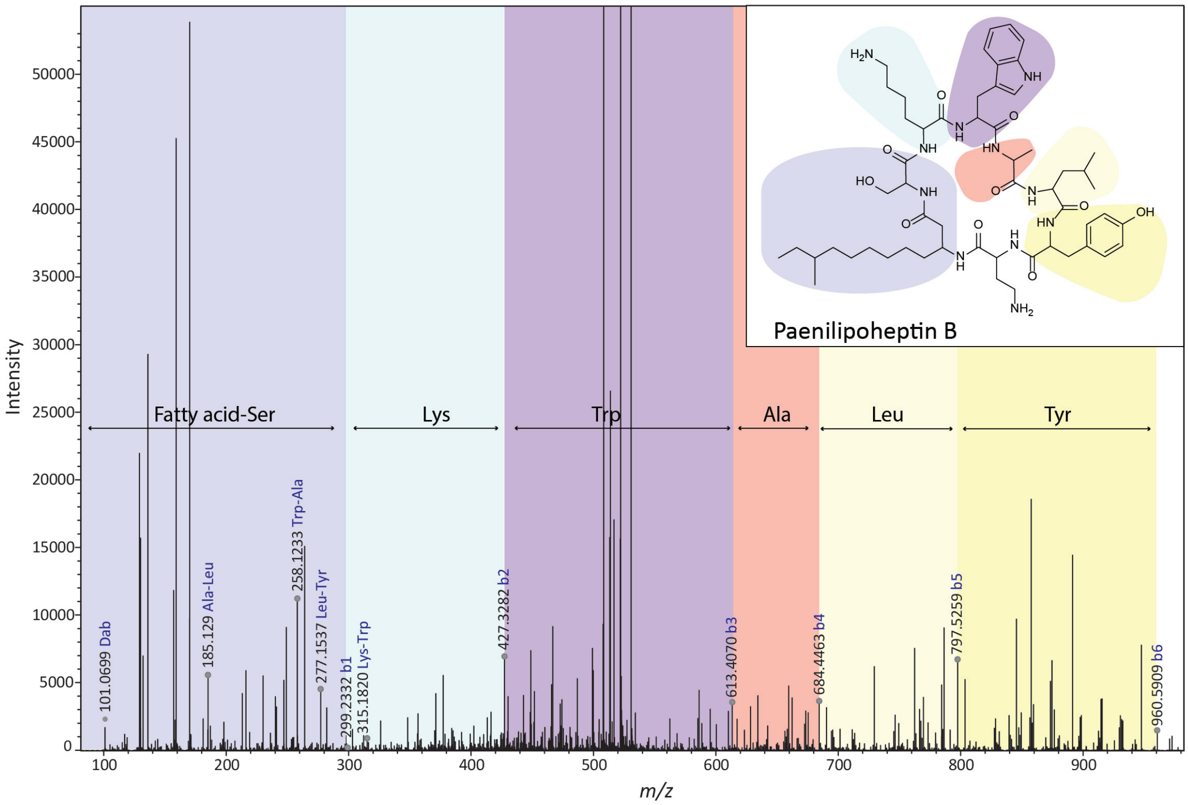

Figure S5. MS/MS spectrum of paenilipoheptin B (precursor ion $[M+2H]^{2+}$ *m/z* 530.8307). The assignment of the sequence of amino acid residues is based on the mass differences between the consecutive *b* ions.

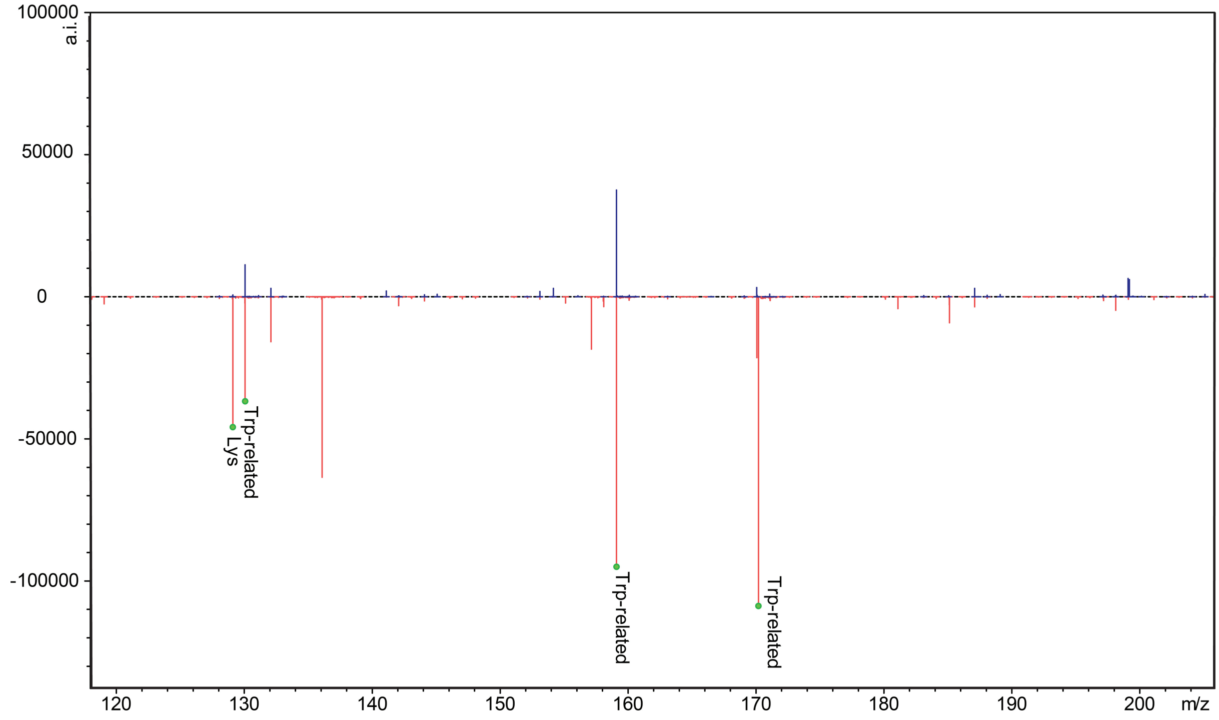

Figure S6. Direct MS/MS spectra comparison of the mass feature with *m/z* 712.8836 (blue) with the mass feature with m/z 530.8307 (red).

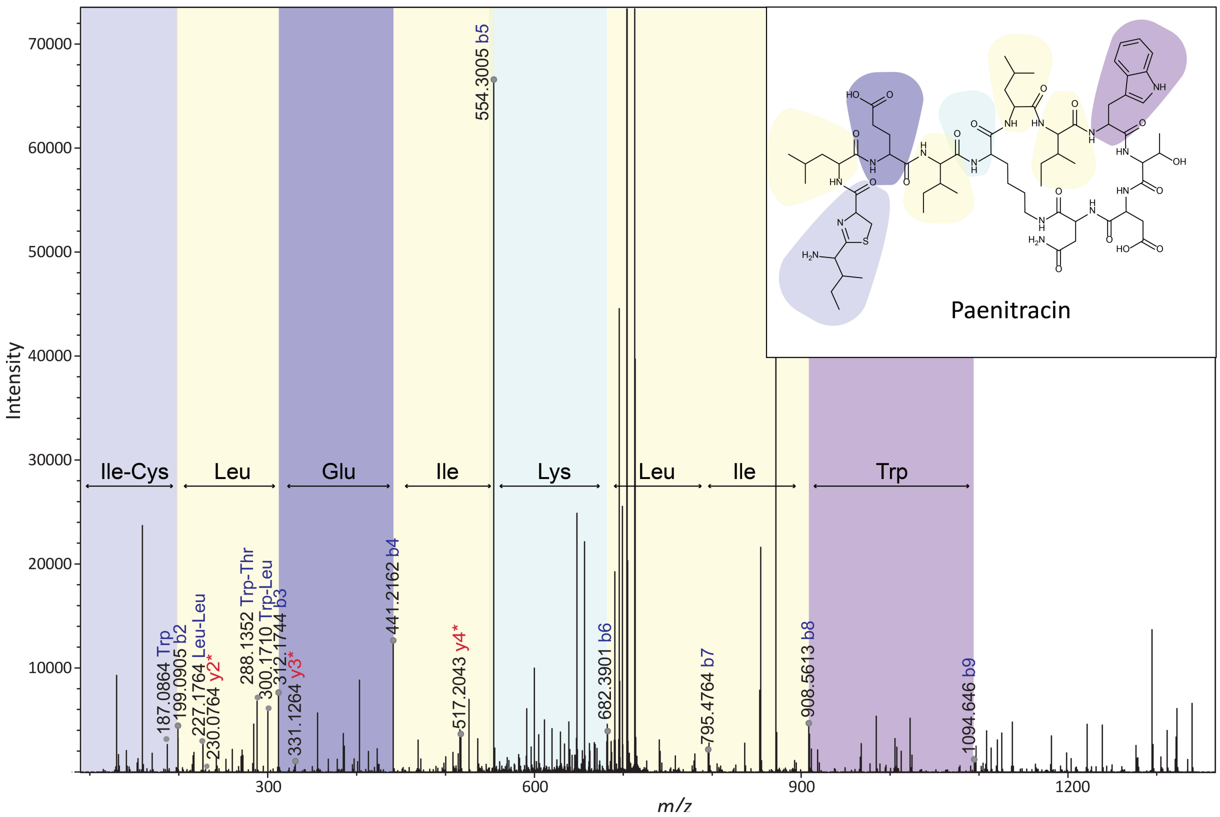

Figure S7. MS/MS spectrum of paenitracin A (precursor ion $[M + 2H]^{2+}$ *m/z* 712.8843). The assignment of the sequence of amino acid residues is based on the mass differences between the consecutive *b* ions.

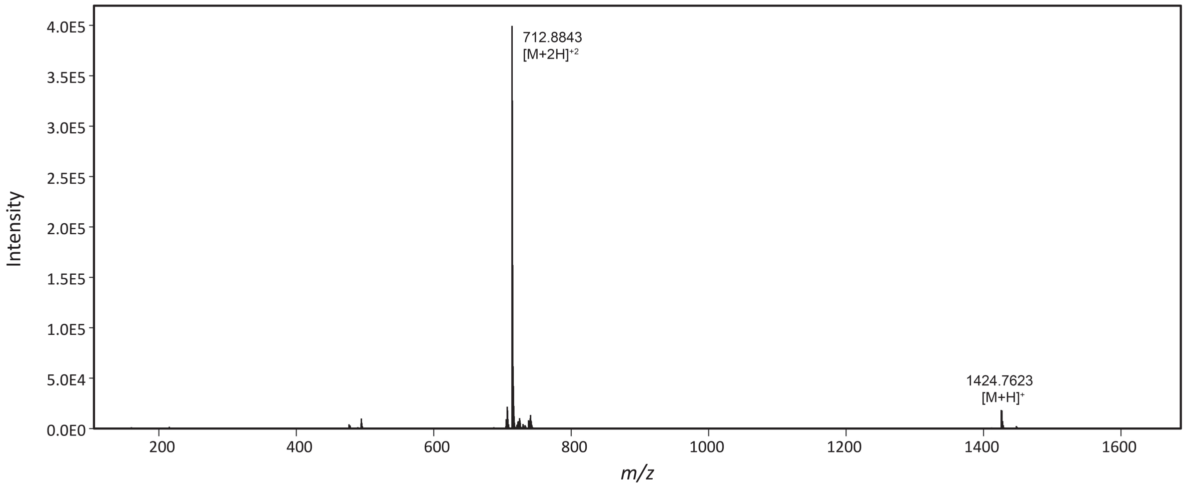

Figure S8. HRMS spectrum of **1**.

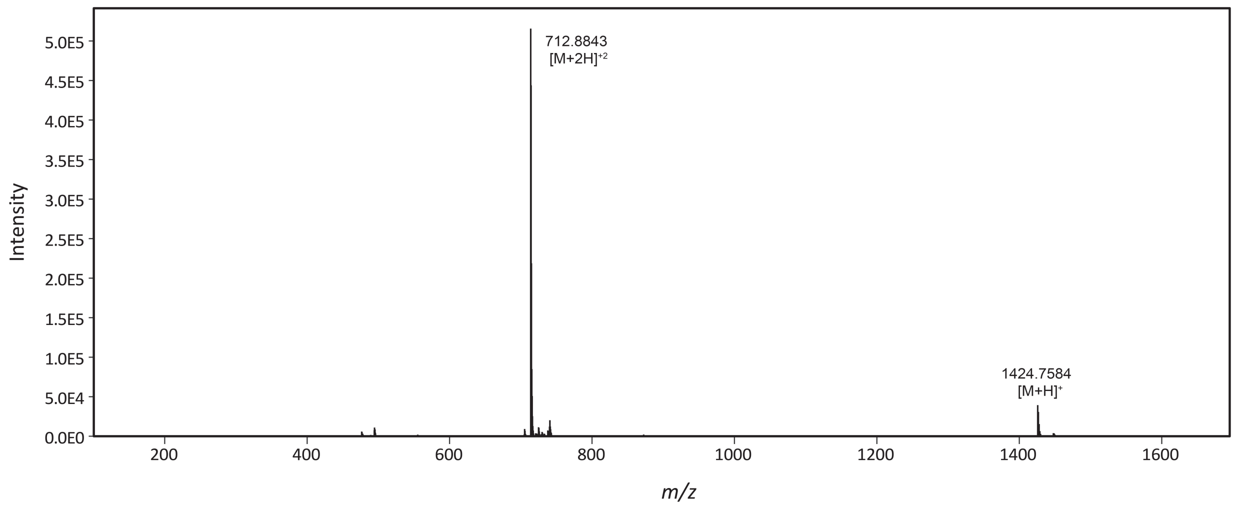

Figure S9. HRMS spectrum of **2**.

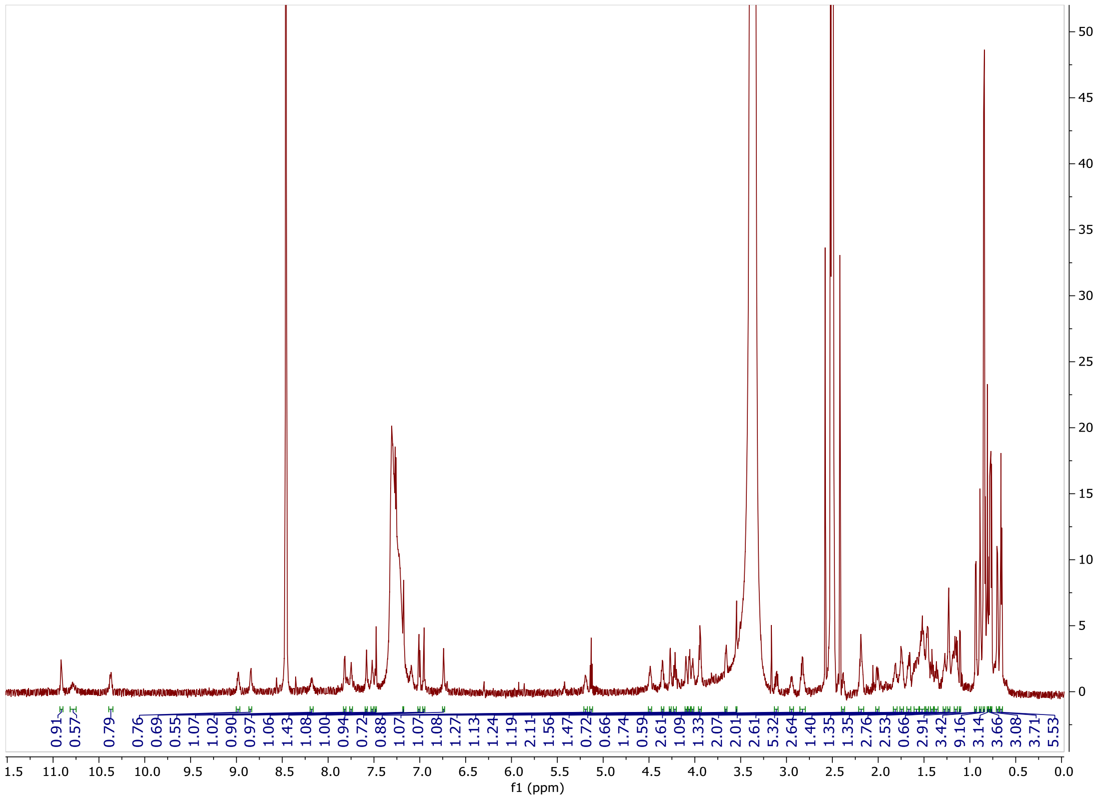

Figure S10. ^{1}H NMR spectrum of **1** (850 MHz, in DMSO-d_6).

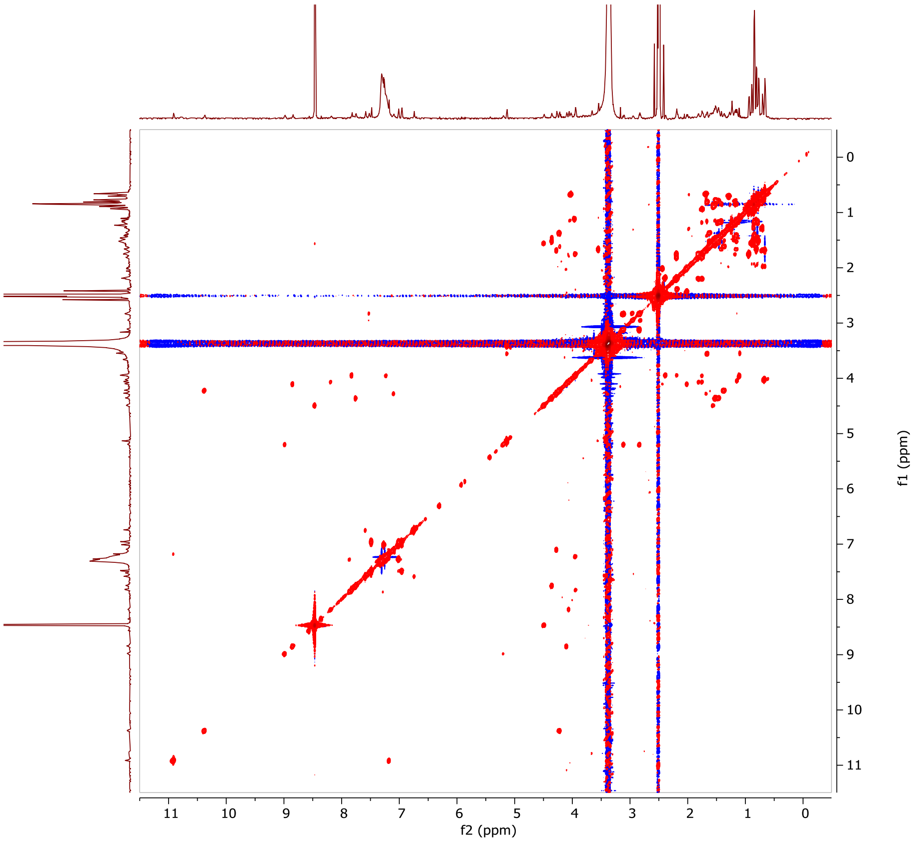

Figure S11. ^{1}H–^{1}H COSY spectrum of **1** (850 MHz, in DMSO-d_6).

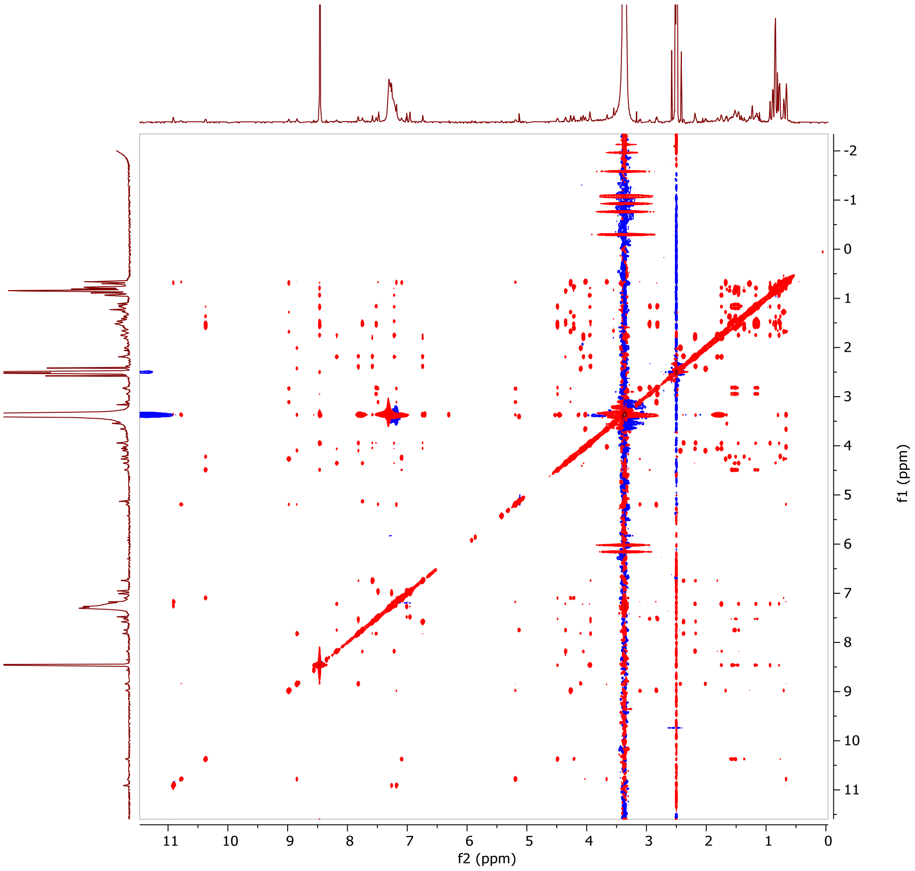

Figure S12. NOESY spectrum of **1** (850 MHz, in DMSO-d_6).

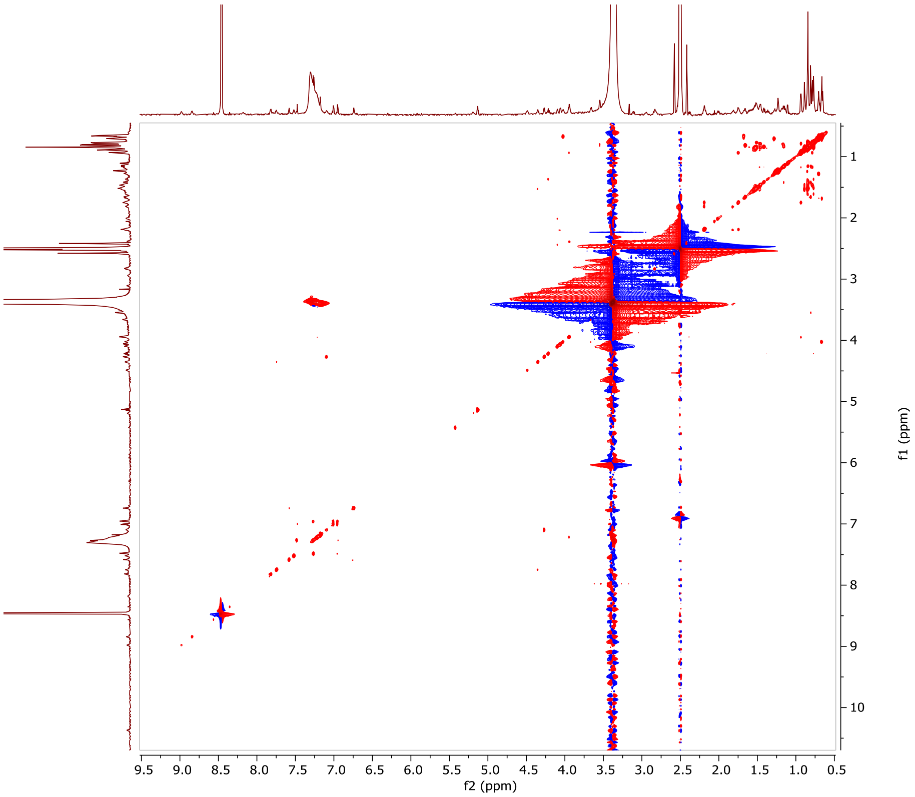

Figure S13. ^{1}H–^{1}H TOCSY spectrum of **1** (850 MHz, in DMSO-d_6).

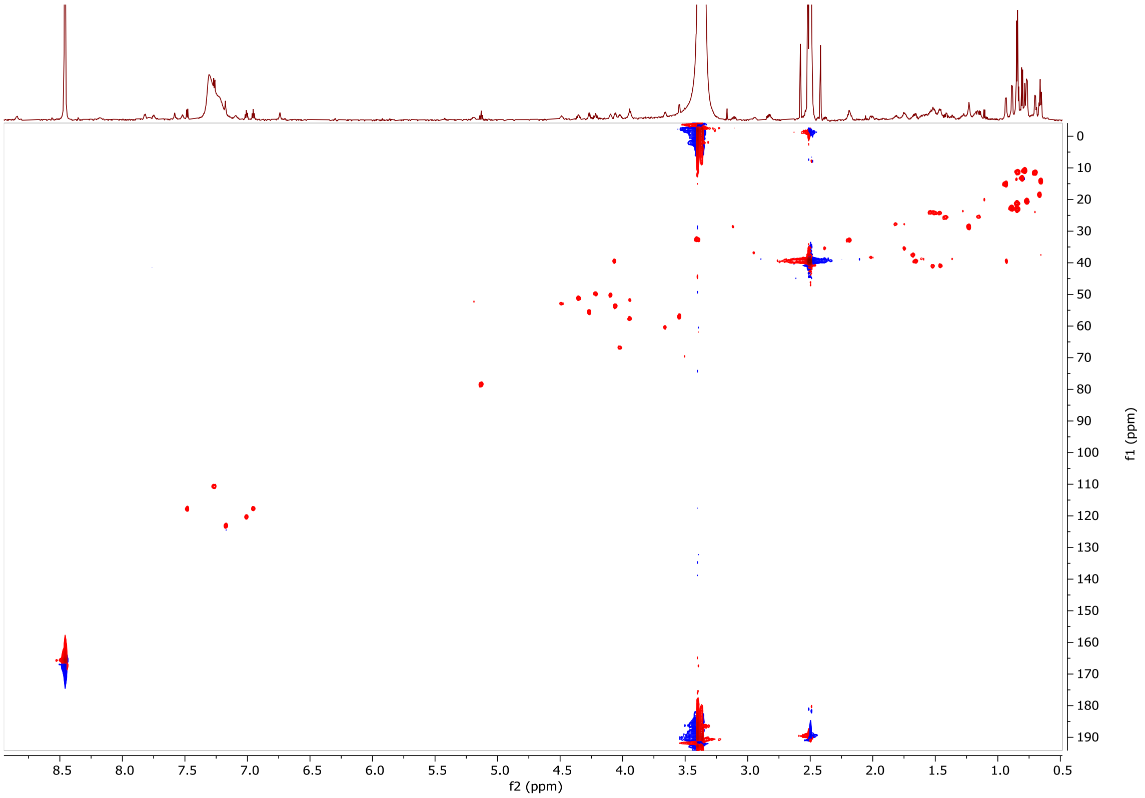

Figure S14. HSQC spectrum of **1** (850 MHz, in DMSO-d_6).

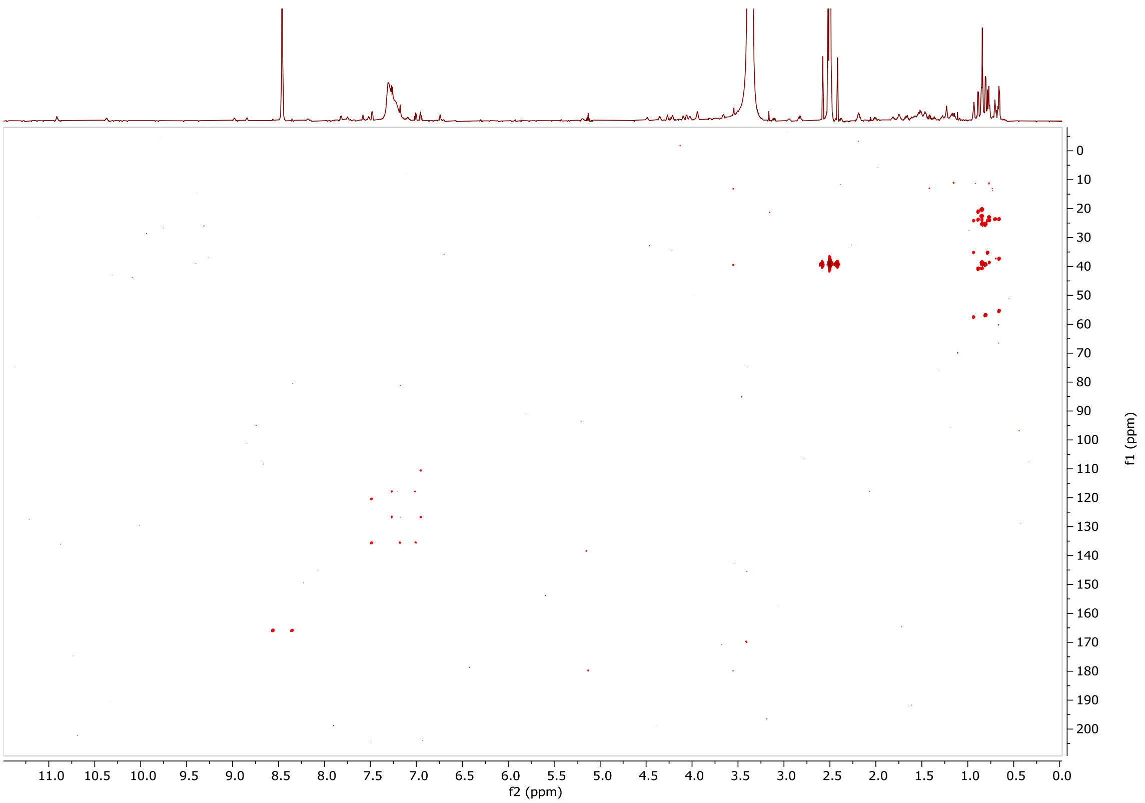

Figure S15. HBMC spectrum of **1** (850 MHz, in DMSO-d_6).

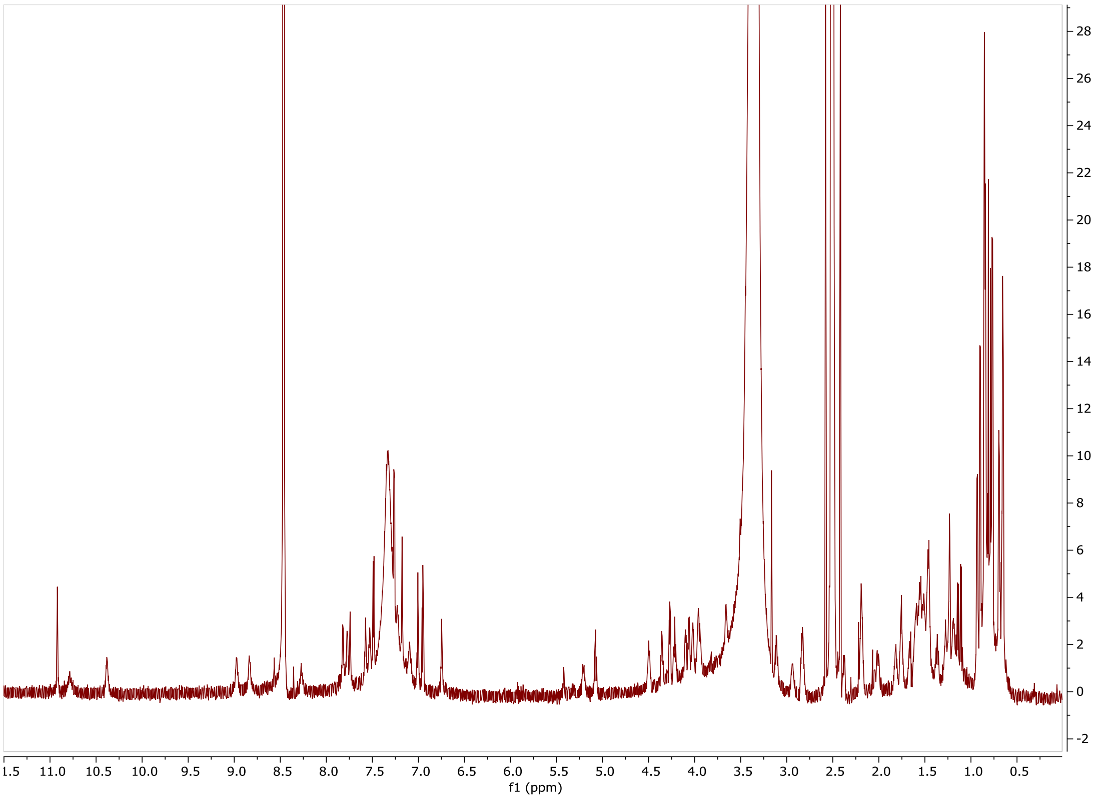

Figure S16. ^{1}H NMR spectrum of **2** (850 MHz, in DMSO-d_6).

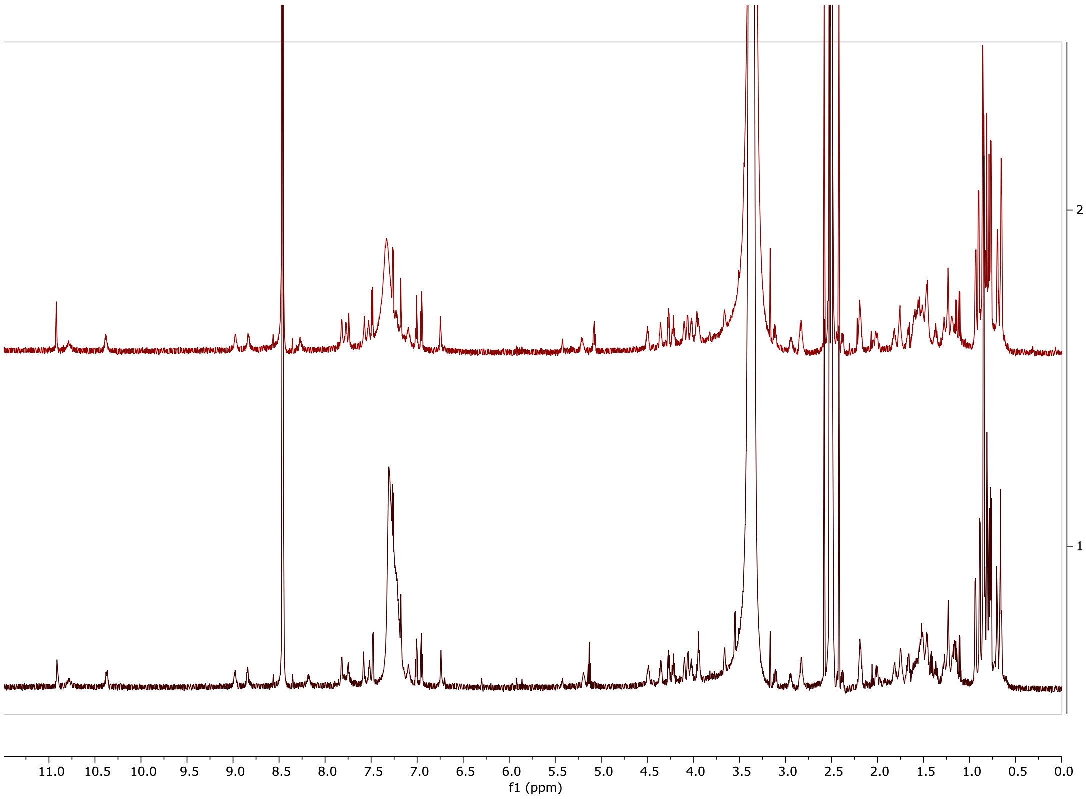

Figure S17. Stacked ^1H NMR spectra of **1** and **2** (850 MHz, in DMSO-d_6).
